# Investigation of mechanisms of mesenchymal stem cells for treatment of diabetic nephropathy via construction of a miRNA-TF-mRNA network

**DOI:** 10.1080/0886022X.2017.1421556

**Published:** 2018-03-13

**Authors:** Hailing Yang, Xiaofei Zhang, Guangda Xin

**Affiliations:** a Department of Emergency, China-Japan Union Hospital of Jilin University, Changchun, China;; b Department of Pediatrics, China-Japan Union Hospital of Jilin University, Changchun, China;; c Department of Nephrology, China-Japan Union Hospital of Jilin University, Changchun, China

**Keywords:** Diabetic nephropathy, differentially expressed gene, protein-protein interaction network, microRNA, transcription factor

## Abstract

**Background:** Recent studies have reported that mesenchymal stem cells (MSCs) exert therapeutic effects on the treatment of diabetic nephropathy (DN), but the underlying mechanisms remain unclear.

**Methods:** A dataset GSE65561 was obtained from Gene Expression Omnibus (GEO) database, which contained four healthy control samples (group 1), four healthy controls samples co-cultured with MSCs (group 2), five DN samples (group 3) and five DN samples co-cultured with MSCs (group 4). The differentially expressed genes (DEGs) between group 3 vs. group 1 and group 4 vs. group 2 were constructed using Linear Models for Microarray (LIMMA) package package. Then, DAVID was used to analyze the functional enrichment of DEGs. Based on STRING database the protein-protein interaction (PPI) network was visualized by the Cytoscape plug-in CytoNCA. Besides, the hub miRNAs and transcription factors (TFs) regulating DEGs were predicted using Webgestalt.

**Results:** Totally, 303 up-regulated and 88 down-regulated DEGs were shared in group 3 vs. group 1 and group 4 vs. group 2. Besides, the up-regulated DEGs were mainly enriched in ‘translation’ and ‘translational elongation’, while the down-regulated genes were only enriched in ‘protein kinase activity’. *RPS27A* and *RPLP0* had a higher degree in the PPI network and they were regulated by *EIF3M*. In addition, *ETF1* was predicted to be an important gene, which was regulated by miR-150, miR-134 and *EIF2S1*.

**Conclusions:*** RPS27A*, *RPLP0* and *ETF1* may be potential targets for MSCs on the treatment of DN.

**Highlights**
- RPS27A and RPLP0 may be important genes in the treatment of MSCs for DN.
- TF EIF3M may play a key role in the treatment of MSCs for DN.
- MiR-150 and miR-134 may be essential microRNAs in the treatment of MSCs for DN.

## Introduction

Diabetic nephropathy (DN) is a progressive nephropathy caused by diabetes mellitus (DM) [1] and about one-third of type 1 DM patients and 25% of type 2 DM develop DN [2,3]. DN is characterized by glomerular hypertrophy, increased renal tubular membrane and glomerular membrane thickness and extracellular matrix accumulation in these membranes, which cause glomerular fibrosis and ultimately lead to damage renal function [4,5]. DN is the main reason for the world’s end-stage renal failure and cardiovascular death [6]. Thus, it has important significance to study the novel treatment strategy for DN.

Infiltration of monocytes and monocyte-derived macrophages is the hallmark of DN [7]. Monocytes play an important role on the defense mechanism of the organism, nevertheless the function of monocytes separated from DN patients have been impaired [8]. CD14 + CD16 + monocytes are related to microinflammation in patients with DN, whose immunological dysfunction are reported to be associated with activation in NF-kappa; B/TLR4 rather than Notch1 signaling pathway [9,10]. Changes in mononuclear cells can reduce inflammation and may contribute to the treatment of patients with type 2 diabetes [9]. Therefore, controlling the numbers of monocytes and macrophages are critical for the treatment of DN.

Mesenchymal stem cells (MSCs) promote the proliferation and maturation of monocytes [11]. Reportedly, human MSCs can modulate the down-regulation of CD8 expression on CD8 + T cells mediated by CD14 + monocytes [12]. MSCs enhance the phagocytic activity of circulating monocytes in a mouse model of gram negative sepsis [13]. In addition, MSCs have been shown to inhibit the initial differentiation of monocyte-derived dendritic cells, thereby ameliorate DN [14]. Moreover, Lv et al. have suggested that MSCs may ameliorate DN through inhibition of MCP-1 expression, reducing macrophages infiltration and down-regulating several proinflammatory factors expression such as IL-6 and TNFα in renal tissue of diabetic rats [15]. However, the mechanisms of MSCs in the treatment for DN have still been partially investigated.

In a recent study, Wise et al. used a microarray analysis to screen differentially expression genes (DEGs) of monocytes before and after MSCs co-culture and the result indicated MSC treatment significantly upregulated genes associated with a reparative macrophage such as cytokines IL-10, IGF1, CCL2 and VEGF-A [11]. However, the regulatory mechanisms of DEGs have not been discussed. The goal of this study was to further screen crucial genes associated with MSCs for treatment of DN by construction of a miRNA-TF-mRNA network using the same dataset of Wise et al. [11]. This study may provide a theoretical basis for the study of MSCs in the treatment of DN.

## Methods

### Microarray data acquisition

The microarray expression data and platform annotation files of GSE65561 [11] uploaded in Gene Expression Omnibus (GEO) database (http://www.ncbi.nlm.nih.gov/geo/query/acc.cgi?acc=GSE65561) were obtained and it was sequenced from the chip platform GPL16686 [HuGene-2_0-st] Affymetrix Human Gene 2.0 ST Array [transcript (gene) version]. A total of 18 cell samples were included in this dataset, four healthy control samples (group 1), four healthy controls samples co-cultured with MSCs for 48 h (group 2), five samples from type 2 diabetic patients with end-stage renal disease groups (group 3) and five samples of diabetic patients with end-stage renal disease groups co-cultured with MSCs for 48 h (group 4). Among them, four healthy control blood samples were obtained from the Australian Red Cross Blood Service and five disease blood samples were obtained from five diabetic patients with end-stage renal disease receiving hemodialysis at the Monash Medical Centre. This study was ethically approved by the Monash Health Human Research Ethics Committee (Monash 10179B) and the Monash University Human Research Ethics Committee (CF07/3495–2007001798). Additionally, informed consent was obtained from participants.

### Identification of DEGs shared in group 3 vs. group 1 and group 4 vs. group 2

The GSE65561 chip data was normalized using the robust multi-array average (RMA) method [16] in the Affy package (Version: 1.52.0) of the R software (Version: 3.3.2). The DEGs in the comparison groups of group 3 vs. group 1 and group 4 vs. group 2 were identified by Linear Models for Microarray (LIMMA) package (Version: 3.30.3) [17] in R software and the t-test was used to calculate *p* values of each DEG. *p* values < .05 was used as the threshold. Additionally, the R package gplots (Version: 3.0.1) was used to draw the DEGs expression heat map, which displayed the clustering of the DEGs in different samples.

### Functional enrichment analysis of DEGs

The Gene Ontology (GO) database stores extensive information of gene sets including GO terms as well as the annotations of genes [18] and the Kyoto Encyclopedia of Genes and Genomes (KEGG) [19] database provides informative pathways for substantial genes. The GO function and KEGG pathway involved in DEGs were analyzed by the online tool DAVID (version: 6.7, https://david-d.ncifcrf.gov/summary.jsp) [20]. The terms with the number of enriched genes count ≥ 2 and the hypergeometric significance *p* values < .05 were considered significant.

### PPI network

Based on the search tool for the retrieval of interacting genes/proteins (STRING) database (Version: 10.0, http://www.string-db.org/) [21], the PPI network of the DEGs was analyzed. The combined score was set to >0.4. Using the Cytoscape plug-in CytoNCA (Version: 2.1.6) [22], the degrees of the three network topological scores were visualized: Degree Centrality (DC), Betweenness centrality (BC) and Closeness centrality (CC) in the network. The parameter was set to ‘network without weight’. Among the CytoNCA output results, the node, edge and degree represent the protein, the relationship pair of proteins and the number of relationship pairs interacted with this protein, respectively. Besides, the nodes with higher degree were considered as the hub proteins [23].

### Sub-module analysis

By using the MCODE plugin of cytoscape (Version: 1.4.2) [24], the sub-module of PPI network was analyzed with the default thresholds: Degree Cutoff: 2; Node Score Cutoff: 0.2; K-Core: 2 and Max; Depth: 100. Besides, the correlation of GO terms for the sub-network module with the highest score were analyzed by using Golorize (Version: 1.0.0.beta1) [25].

### Prediction of regulation relationship between miRNAs and target genes

The miRNAs that targeted the DEGs were predicted using Webgestalt (http://www.webgestalt.org/) [26]. The number of enriched genes ≥2 and adjusted *p* < .05 were chosen as the threshold. Moreover, the hypergeometric test and Benjamini-Hochberg (BH) [27] adjustment were conducted.

### Prediction of TFs

Based on the transcriptional regulatory network data in the ITFP (http://itfp.biosino.org/itfp) and TRANSFAC (http://www.gene-regulation.com/pub/databases.html) databases, the TFs in DEGs were predicted and then the DEGs regulated by these TFs were identified.

## Results

### Screening of DEGs

**Figure 1.**
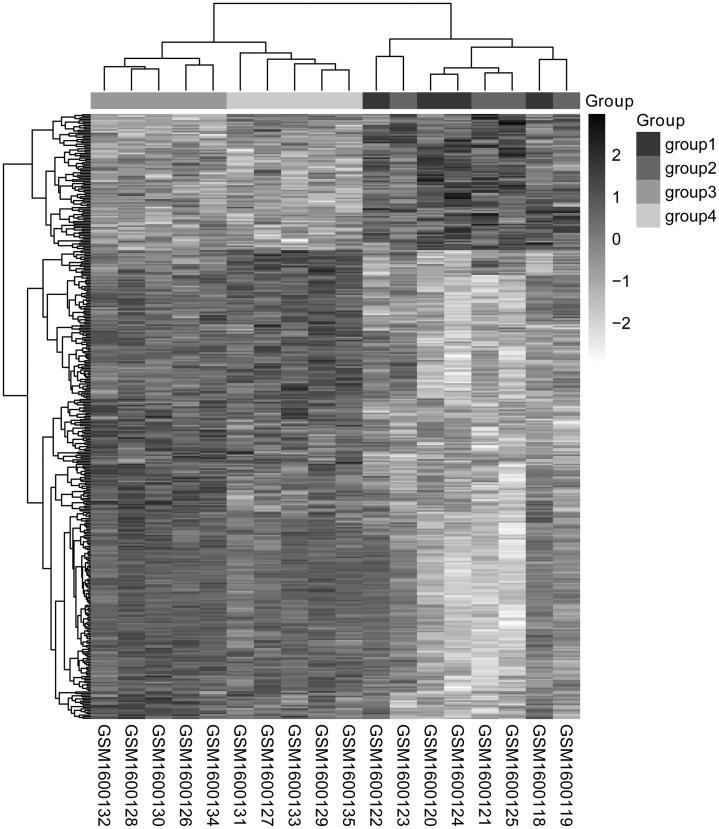
Two-way hierarchical clustering heat map of DEGs. The horizontal axis represents the different samples, the vertical axis represents the genes and the white to black represents the genes expression changed; the deeper the color, the high the expression levels. DEGs: differentially expressed genes.

A total of 1622 DEGs including 916 up-regulated genes and 706 down-regulated genes were screened from comparison groups of group 3 vs. group 1. And there were 1289 up-regulated DEGs and 1112 down-regulated DEGs in comparison groups of group 4 vs. group 2. Additionally, the overlapped genes between these two comparison groups were obtained, 303 genes were finally found to be up-regulated and 88 genes were down-regulated in group 3 and 4 compared to group 1 and 2, respectively. These DEGs could well distinguish from the four kinds of samples based on the DEGs expression heat map (Figure 1).

### Functional enrichment analysis of the DEGs

To explore potential function and pathways for these DEGs, GO functional and KEGG pathway enrichment of both up- and down-regulated DEGs were analyzed. The up-regulated DEGs were mainly enriched in functional or pathway categories such as “translation (e.g., *RPS27A* and *RPLP0*)”, “translational elongation (e.g., *RPS27A* and *RPLP0*)”, ‘ribosome (e.g., *RPS27A*)’, ‘ribonucleoprotein complex (e.g., *RPS27A*)’, ‘structural constituent of ribosome (e.g., *RPS27A*)’, ‘Parkinson’s disease (e.g., *NDUFB3*)’ and ‘oxidative phosphorylation (e.g., *ATP5G3*)’. The down-regulated genes were mainly enriched in ‘protein kinase activity (e.g., *MAP3K12*)’ (Table 1).

**Table 1.** Results of enrichment analysis of DEGs.

Terms	Count	*p* value	Genes
Up-regulated genes enriching BP			
GO:0006412∼translation	33	1.23E-16	*MRPS16*, *PABPC4*, *MRPS10*, *RPS15A*, *RPL13AP20*, *DTD1*, *RPL21P28*, *RPS3*, *MRPL11*, *EIF3G*, *RPL9*, *RPLP0*, *EIF1AY*, *RPL26L1*, *MRPL37*, *EIF3I*, *EIF3M*, *RPS27A*, *MRPL35*, *RPL36AL*, *EEF1A1*, *RPSAP19*, *RPL27*, *RPS9*, *MRPS21*, *MRPS7*, *ETF1*, *RPS5*, *RPS19*, *MRPL27*, *RPS14*, *EIF2S1*, *MRPL47*
GO:0006414∼translational elongation	14	6.47E-9	*EEF1A1*, *RPSAP19*, *RPS9*, *RPL27*, *RPS15A*, *RPS5*, *RPS3*, *RPL21P28*, *RPS19*, *RPLP0*, *RPL9*, *RPS14*, *RPL26L1*, *RPS27A*
GO:0006119∼oxidative phosphorylation	12	4.03E-7	*NDUFB3*, *NDUFB5*, *UQCR11*, *NDUFB7*, *NDUFAB1*, *ATP5F1*, *NDUFS2*, *ATP5H*, *ATP5G3*, *NDUFS1*, *ATP6V1F*, *UQCRHL*
GO:0031397∼negative regulation of protein ubiquitination	10	2.38E-6	*PSMB7*, *GTPBP4*, *PSMB6*, *PSMC5*, *PSMB1*, *PSMA5*, *PSME3*, *PSMB8*, *RPS27A*, *PSMB9*
GO:0031400∼negative regulation of protein modification process	12	2.84E-6	*PSMB7*, *GTPBP4*, *PSMB6*, *PSMC5*, *PSMB1*, *PSMA5*, *NLRP12*, *PEBP1*, *PSME3*, *PSMB8*, *RPS27A*, *PSMB9*
Up-regulated genes enriching CC			
GO:0005840∼ribosome	27	3.98E-16	*MRPS16*, *MRPS10*, *RPS15A*, *RPL13AP20*, *RPL21P28*, *RPS3*, *MRPL11*, *RPLP0*, *RPL9*, *RPL26L1*, *MRPL54*, *MRPL37*, *RPS27A*, *MRPL35*, *RPL36AL*, *RPSAP19*, *MRPS23*, *RPS9*, *RPL27*, *MRPS21*, *MRPS7*, *RPS5*, *RPS19*, *MRPL27*, *RPS14*, *MRPL47*, *MRPL46*
GO:0030529∼ribonucleoprotein complex	38	5.29E-15	*MRPS16*, *STRAP*, *PABPC4*, *CWC15*, *SNRPD1*, *MRPS10*, *RPS15A*, *RPL13AP20*, *SF3B4*, *RPL21P28*, *RPS3*, *MRPL11*, *RPL9*, *RPLP0*, *RPL26L1*, *MRPL54*, *PPIL3*, *MRPL37*, *LSM3*, *RPS27A*, *MRPL35*, *RPL36AL*, *BCAS2*, *TXNL4B*, *RPSAP19*, *MRPS23*, *RPL27*, *RPS9*, *MRPS21*, *MRPS7*, *RPS5*, *RPS19*, *MRPL27*, *RPS14*, *EIF2S1*, *MRPL47*, *POP4*, *MRPL46*
GO:0044429∼mitochondrial part	39	9.30E-14	*NDUFB3*, *MRPS16*, *NDUFB5*, *NDUFB7*, *COX7B*, *NDUFAB1*, *TIMM10*, *ECHS1*, *TIMM50*, *TIMM13*, *SFXN2*, *COX5A*, *ATP5G3*, *DTD1*, *MRPL11*, *ACSL1*, *UQCR11*, *PARL*, *COX6B1*, *MRPL37*, *ATP5H*, *NDUFS2*, *NDUFS1*, *MRPL35*, *ACAA2*, *SLC25A5*, *SLC25A6*, *ATP5F1*, *COX4I1*, *MRPS21*, *TIMM8B*, *UQCRHL*, *PPIF*, *MRPL27*, *PEBP1*, *MRPL47*, *TOMM22*, *PMPCA*, *SURF1*
GO:0005743∼mitochondrial inner membrane	28	2.97E-13	*NDUFB3*, *NDUFB5*, *NDUFB7*, *COX7B*, *TIMM10*, *NDUFAB1*, *TIMM50*, *TIMM13*, *SFXN2*, *COX5A*, *ATP5G3*, *UQCR11*, *PARL*, *COX6B1*, *NDUFS2*, *ATP5H*, *NDUFS1*, *ACAA2*, *SLC25A5*, *SLC25A6*, *ATP5F1*, *COX4I1*, *TIMM8B*, *UQCRHL*, *PPIF*, *TOMM22*, *SURF1*, *PMPCA*
GO:0005739∼mitochondrion	52	5.69E-13	*GM2A*, *NDUFAB1*, *TIMM50*, *COX5A*, *C14ORF2*, *MTHFD2*, *UQCR11*, *PARL*, *MRPL37*, *ATP5H*, *NDUFS2*, *NDUFS1*, *MRPL35*, *ACAA2*, *SLC25A5*, *SLC25A6*, *COX4I1*, *MRPS7*, *TIMM8B*, *TMEM186*, *PEBP1*, *MRPL47*, *TOMM22*, *PMPCA*, *SURF1*, *MRPL46*, *NDUFB3*, *MRPS16*, *NDUFB5*, *NAPG*, *NDUFB7*, *COX7B*, *TIMM10*, *MRPS10*, *ECHS1*, *CHCHD2*, *TIMM13*, *SFXN2*, *ATP5G3*, *DTD1*, *MRPL11*, *ACSL1*, *MRPL54*, *COX6B1*, *MRPS23*, *ATP5F1*, *MRPS21*, *UQCRHL*, *PPIF*, *MRPL27*, *ALKBH3*, *VPS25*
Up-regulated genes enriching MF			
GO:0003735∼structural constituent of ribosome	24	5.92E-16	*RPSAP19*, *MRPS16*, *MRPS10*, *RPS15A*, *RPS9*, *RPL27*, *MRPS21*, *RPL13AP20*, *MRPS7*, *RPS5*, *RPS3*, *RPL21P28*, *MRPL11*, *RPS19*, *MRPL27*, *RPS14*, *RPL9*, *RPLP0*, *RPL26L1*, *MRPL37*, *MRPL47*, *RPS27A*, *MRPL35*, *RPL36AL*
GO:0015078∼hydrogenion transmembrane transporter activity	11	9.80E-7	*UQCR11*, *COX7B*, *COX6B1*, *ATP5F1*, *COX4I1*, *COX5A*, *SURF1*, *ATP5H*, *ATP5G3*, *ATP6V1F*, *UQCRHL*
GO:0005198∼structural molecule activity	28	1.02E-6	*MRPS16*, *MRPS10*, *RPS15A*, *RPL13AP20*, *TPM4*, *RPL21P28*, *RPS3*, *MRPL11*, *RPLP0*, *RPL9*, *RPL26L1*, *TUBB6*, *MRPL37*, *RPS27A*, *MRPL35*, *RPL36AL*, *RPSAP19*, *RPS9*, *RPL27*, *MRPS21*, *MRPS7*, *EMILIN2*, *RPS5*, *RPS19*, *MRPL27*, *RPS14*, *MRPL47*, *LAMC1*
GO:0003723∼RNA binding	29	3.53E-6	*CNBP*, *PABPC4*, *CWC15*, *SNRPD1*, *RPS15A*, *TIMM50*, *SF3B4*, *RPL21P28*, *RPS3*, *MRPL11*, *EIF3G*, *RPLP0*, *RPL9*, *NUDT21*, *EIF1AY*, *LSM3*, *NXF3*, *RPS9*, *MRPS7*, *ETF1*, *CRYZ*, *RPS5*, *RPS19*, *ABT1*, *RPS14*, *EIF2S1*, *PSPC1*, *CIRBP*, *POP4*
GO:0015077∼monovalent inorganic cation transmembrane transporter activity	11	3.73E-6	*UQCR11*, *COX7B*, *COX6B1*, *ATP5F1*, *COX4I1*, *COX5A*, *SURF1*, *ATP5H*, *ATP5G3*, *ATP6V1F*, *UQCRHL*
Up-regulated genes enriching pathway			
hsa05012:Parkinson's disease	17	1.96E-9	*NDUFB3*, *NDUFB5*, *NDUFB7*, *SLC25A5*, *SLC25A6*, *COX7B*, *ATP5F1*, *NDUFAB1*, *COX4I1*, *COX5A*, *ATP5G3*, *UQCRHL*, *UQCR11*, *COX6B1*, *NDUFS2*, *ATP5H*, *NDUFS1*
hsa00190:Oxidative phosphorylation	16	2.02E-8	*NDUFB3*, *NDUFB5*, *NDUFB7*, *COX7B*, *ATP5F1*, *NDUFAB1*, *COX4I1*, *COX5A*, *ATP5G3*, *UQCRHL*, *ATP6V1F*, *UQCR11*, *COX6B1*, *NDUFS2*, *ATP5H*, *NDUFS1*
hsa05016:Huntington's disease	18	4.42E-8	*NDUFB3*, *NDUFB5*, *NDUFB7*, *SLC25A5*, *SLC25A6*, *AP2S1*, *COX7B*, *ATP5F1*, *NDUFAB1*, *COX4I1*, *COX5A*, *ATP5G3*, *UQCRHL*, *UQCR11*, *COX6B1*, *NDUFS2*, *ATP5H*, *NDUFS1*
hsa03010:Ribosome	13	8.01E-8	*RPSAP19*, *RPS9*, *RPL27*, *RPS15A*, *RPS5*, *RPS3*, *RPL21P28*, *RPS19*, *RPLP0*, *RPL9*, *RPL26L1*, *RPS27A*, *RPL36AL*
hsa05010:Alzheimer's disease	15	2.47E-6	*NDUFB3*, *NDUFB5*, *NDUFB7*, *COX7B*, *ATP5F1*, *NDUFAB1*, *COX4I1*, *COX5A*, *ATP5G3*, *UQCRHL*, *UQCR11*, *COX6B1*, *NDUFS2*, *ATP5H*, *NDUFS1*
Down-regulated genes			
GO:0004672∼protein kinase activity	6	0.037	*TYK2*, *FASTKD1*, *ITGAE*, *PDK4*, *MAP3K8*, *MAP3K12*

DEGs: differentially expressed genes; BP: biological process; CC: cell component; MF: molecular function; GO: gene ontology; KEGG: Kyoto Encyclopedia of Genes and Genomes

### Analysis of PPI network

**Figure 2.**
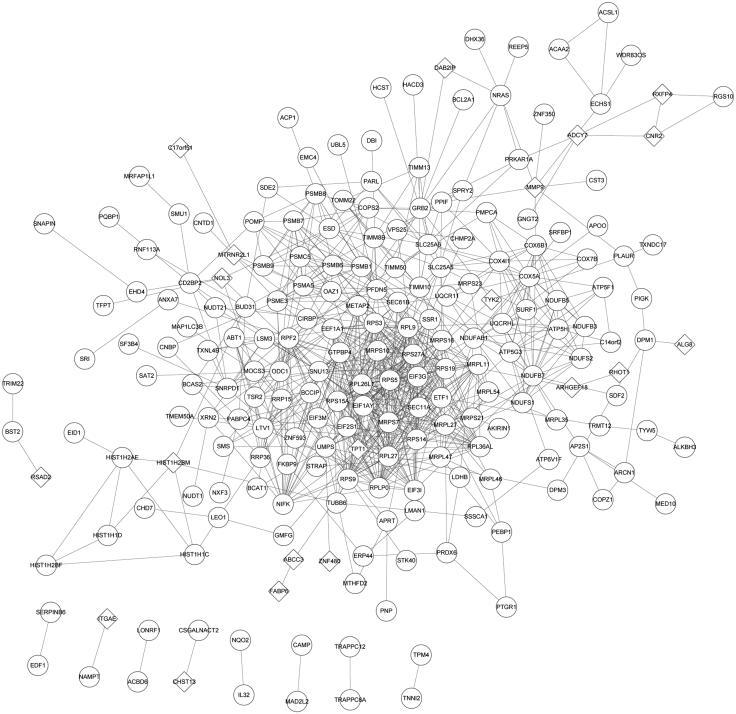
The PPI network of DEGs. Round represents the upregulated DEG; quadrilateral represents down-regulated DEG and the line represents the interaction. DEG: differentially expressed gene.

To determine the interaction relationship between the proteins expressed by these DEGs, a PPI network was constructed. A total of 216 nodes (20 down-regulated and 196 up-regulated) and 790 PPI pairs composed the PPI network (Figure 2). Among them, there were 18 nodes with node degree greater than 20 and all of them (such as *RPS27A* and *RPLP0)* were up-regulated genes (Table 2).

**Table 2.** Genes with node degree greater than 20 in PPI network.

Gene	Degree	Betweenness	Closeness
RPS27A	37.0	3961.025	0.046830755
RPLP0	34.0	1496.081	0.046871595
RPL26L1	34.0	962.4223	0.04664786
RPS3	34.0	1346.5127	0.046749294
RPL9	32.0	980.9833	0.046718817
RPS15A	31.0	689.46735	0.046769634
RPS14	30.0	1155.3206	0.04668838
RPS5	30.0	1379.4094	0.046728972
RPL27	30.0	888.37836	0.046597313
SNU13	29.0	4506.537	0.04646639
RPS19	27.0	831.52124	0.046718817
MRPS7	26.0	2373.208	0.04678999
EEF1A1	24.0	3594.4336	0.046830755
RPS9	24.0	167.19829	0.04642626
MRPS10	23.0	181.42064	0.046406217
NIFK	23.0	1317.9271	0.046306267
MRPL11	21.0	285.20667	0.04619682
RPL36AL	21.0	237.57062	0.04621668

PPI: protein–protein interaction

### Sub-network module mining

**Figure 3.**
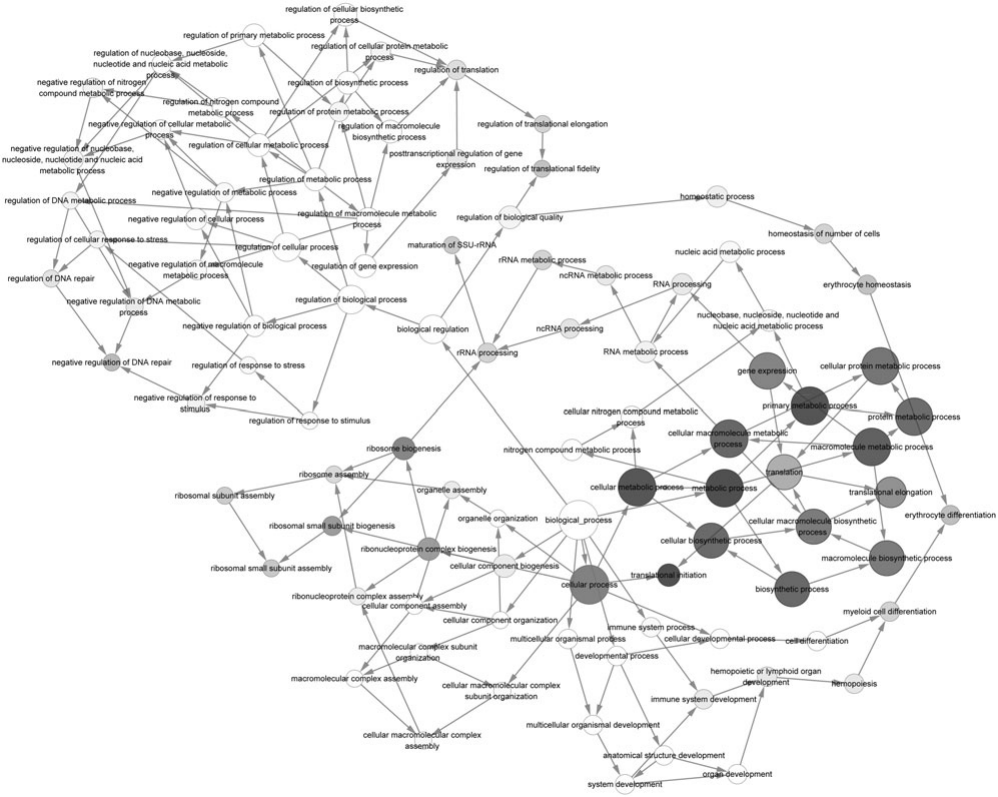
The GO correlation of highest score sub-modules. Gray nodes represent the significant GO terms and white nodes represent the non-significant GO terms. Node in a larger node size and a darker color represents a more significant GO term; arrows indicate relevancy of the two GO terms. GO: gene ontology.

**Figure 4.**
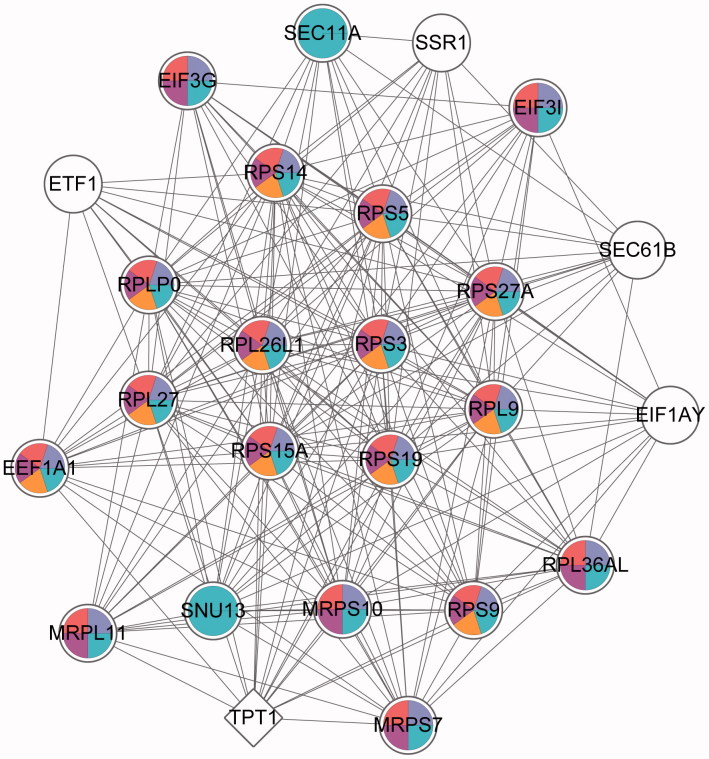
GO enrichment of the highest score sub-modules. Round nodes represent the upregulated genes; quadrilateral nodes represent down-regulated genes and the lines represent the interactions between the two nodes. White nodes represent these genes that are not significantly enriched in the pathway; the different colors are marked as specific GO classifications, blue: gene expression; purple: cellular macromolecule bio synthetic process; red: macromolecule biosynthetic process; rose red: translation and orange: translational elongation. GO: gene ontology (refer online version for color figure).

The sub-network of PPI network was discovered. According to the predefined criterion, 13 sub-network modules were obtained. Among them, the module with the highest score (19.167) contained 25 nodes and 230 edges (regulatory relationship). To understand the potential function of these sub-module DEGs, the GOlorize plug-in was used to analyze the correlation of GO terms with the highest-scoring module. The genes in the highest-scoring module were mainly enriched in the pathways associated with ‘translation and translational elongation’ (Figure 3). Besides, the ‘translation’ was related to ‘translational elongation’. In order to understand the enrichment of genes in the highest-scoring module, we analyzed the enrichment of highest-scoring module genes in the top five GO terms (Figure 4). It was found that 20 nodes enriched in ‘gene expression’ (e.g., *RPS27A* and *RPLP0*), 18 nodes enriched in ‘cellular macromolecule bio synthetic process genes’, ‘macromolecule biosynthetic process’ and ‘translation’ (e.g., *RPS27A* and *RPLP0*) and 12 nodes were enriched in ‘translational elongation’ (e.g., *RPS27A* and *RPLP0*), while only five of them were not enriched in any of them.

### Prediction of miRNA regulated target genes relationship

**Figure 5.**
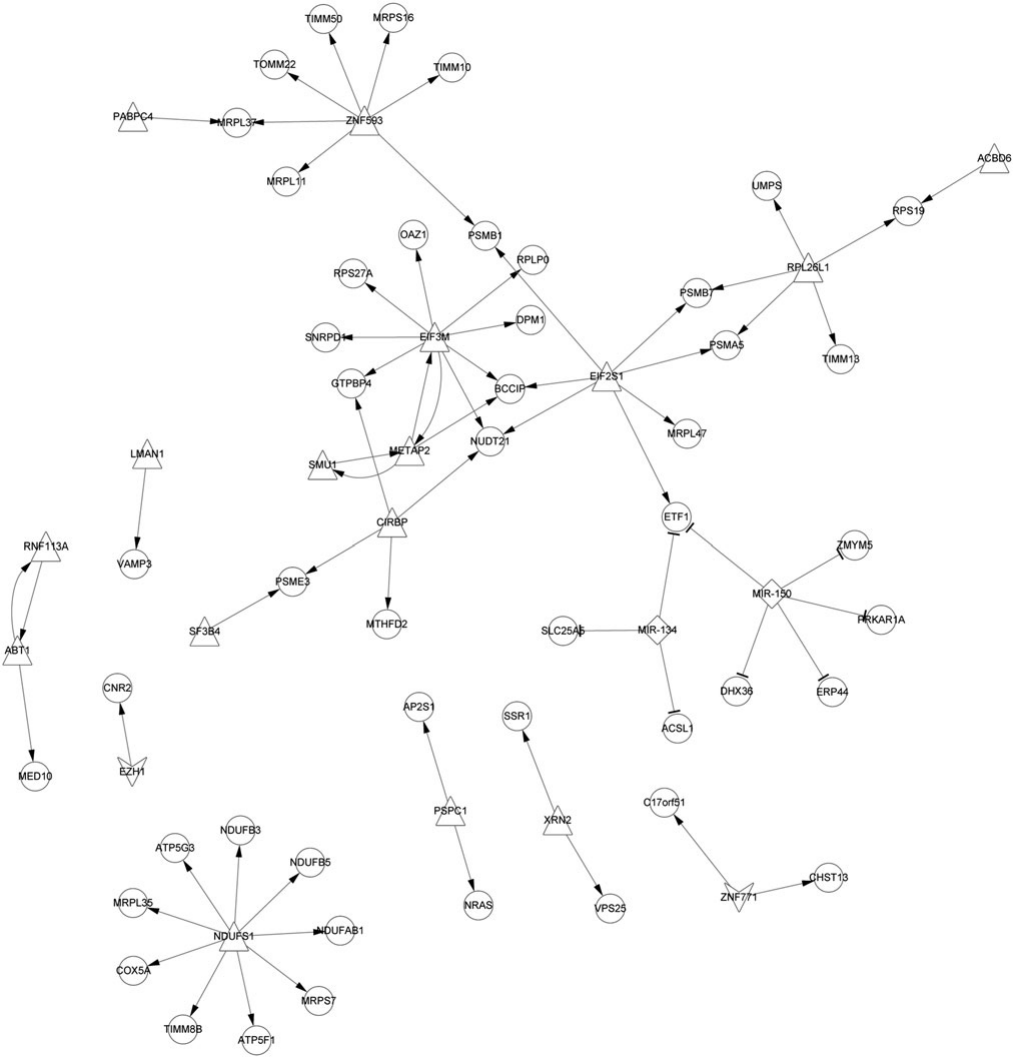
MiRNA-TF-DEGs regulatory network. The triangle nodes represent the up-regulated TFs; the quadrilateral nodes represent the down-regulated TFs; the arrow is the regulation relation; the circle nodes represent the target genesand diamond is the miRNA. DEG: differentially expressed gene; TF: transcription factor.

Prediction of miRNAs was aimed to further understand which miRNAs regulate these DEGs. According to the preset conditions, two miRNAs were selected: hsa-miR-150 (*p* = .0073) and hsa-miR-134 (*p* = .0280). Hsa-miR-150 and hsa-miR-134 were predicted to regulate five genes (*ERP44*, *ETF1*, *DHX36*, *ZMYM5* and *PRKAR1A*) and three DEGs (*ETF1*, *ACSL1* and *SLC25A5*), respectively (Figure 5).

### Prediction of TFs

TFs play an important role in the regulation of gene expression, so the TFs that regulated DEGs were also predicted. A total of 16 (e.g., *EIF3M*, *METAP2* and *SMU1*) and two (*EZH1* and *ZNF771*) TFs were predicted to regulate the up-regulated genes and down-regulated DEGs, respectively. For example, NDUFS1 regulated nine DEGs (e.g., *ATP5G3*); *EIF3M* regulated eight DEGs (e.g., *BCCIP* and *NUDT21*); seven DEGs (e.g., *PSMB1*) regulated by ZNF593 (Figure 5).

## Discussion

Our study explored the DEGs of monocytes isolated from control and DN which were co-cultured with and without MSCs and predicted the miRNAs and TFs which targeted these DEGs. Totally, 303 up-regulated and 88 down-regulated DEGs shared in group 3 vs. group 1 and group 4 vs. group 2. Beside, *RPS27A* (ribosomal protein S27a) and *RPLP0* (ribosomal protein lateral stalk subunit P0) had a higher degree in the PPI network and they were regulated by *EIF3M* (eukaryotic translation initiation factor 3 subunit M). In addition, *ETF1* (eukaryotic translation termination factor 1) was predicted to be an important gene, which was regulated by miR-150, miR-134 and *EIF2S1* (eukaryotic translation initiation factor 2 subunit alpha).

MSCs promote the proliferation of monocytes, which contribute to the repair of macrophages on renal fibrosis [11]. DN causes renal fibrosis and ultimately lead to decreased renal function [5]. The increased numbers of circulating monocytes in type 2 diabetes mellitus display pro-inflammatory properties and promote the secretion of pro-inflammatory cytokines [9]. It has been reported that MSCs display a more pronounced immunomodulatory effects in an inflammatory environment than steady-state, whereby the immunosuppressive properties of MSCs are caused by inflammatory stimuli [28,29]. Furthermore, *RPS27A* regulates cells cycle arrest through p53, Raf/MEK/ERK, P21 and BCL-2 signaling pathways, promotes cell proliferation and inhibits cell apoptosis [30]. This was consistent with our results that the expression of *RPS27A* which was targeted by *EIF3M* in monocytes was up-regulated after MSCs co-cultured. *EIF3M* binds to tumor-associated genes in human colon cancer cell lines, which in turn affects cell proliferation, cell cycleand cell apoptosis [31]. *RPLP0* which is an integral part of the ribosomal 60 S subunit was also regulated by *EIF3M*. In gastric cancer, *RPLP0* deficiency can suppress cells growth and cells cycle by down-regulating *CDK2* [32]. *RPLP0* also affects the expression of p21 and has anti-apoptotic effect on gastric cancer [32]. However, the role of *EIF3M* and *RPLP0* in DN remains unclear. The cell numbers of monocytes isolated from control and DN subjects were less than that of cultured with MSCs [11]. Taken together, we suggested that *RPS27A* and *RPLP0* may be the key genes in MSCs treatment for DN mononuclear cells. During this process, *EIF3M* may interact with *RPS27A* and *RPLP0* to regulate their expression and promote cell proliferation.

MiRNAs, a small non-coding RNA of about 19–26 nt, play an important role in cell proliferation, apoptosis, cell differentiation and tumorigenesis [33]. MiRNAs affect renal fibrosis in kidney disease, among which miR-150 promotes renal fibrosis through down-regulating *SOCS1* [34]. MiR-150 is a biomarker of renal injury in lupus nephritis [34]. In this study, hsa-miR-150 was predicted to target DEGs, such as *ETF1*, which interacted with *RPS27A* and *RPLP0*. Additionally, hsa-miR-134 was also predicted to regulate *ETF1*. MiR-134 affects the cell adhesion of MSCs by mediating the expression of β1 integrin [35] and inhibits cell proliferation via targeting KRAS in human renal cell carcinoma cells [36]. In conclusion, miR-150 and miR-134 were predicted to be the important miRNAs in the treatment of MSCs for DN. They may regulate the expression of *RPS27A* and *RPLP0* by interacting with *ETF1* and thereby promote the proliferation of monocytes in the process.

Andrea et al. [11] have found that, the numbers of up-regulated and down-regulated DEGs shared by group 3 vs. group 1 and group 4 vs. group 2 were 212 and 112 , respectively which was different from our results. The difference may be caused by that in their study, the normalization of data are conducted by bio-conductor packages in R, while we used Affy package of R software to normalize the chip data in the present study. In addition, in order to screen more DEGs, the threshold value of *p* values < .05 was applied, whereas the thresholds for screening DEGs in their study were set as |log_2_ fold change| ≥ 0.75 and the false discovery rate of revised *p* values ≤ .05.

In short, *RPS27A*, *RPLP0* and *ETF1* may be the potential target gene for the treatment of MSCs in DN, which provide a theoretical basis for the investigation of MSCs in the treatment of DN. However, these results were not validated via experiment and their specific pathways of regulation were not been studied, so future studies can focus on the research of specific pathways via experiment.
